# Supplementary material for: Opioid agonist treatment and risk of death or rehospitalization following injection drug use–associated bacterial and fungal infections: A cohort study in New South Wales, Australia
Source: PLoS Med. 2022 Jul 19;19(7):e1004049. doi: 10.1371/journal.pmed.1004049 (PMC9295981; doi:10.1371/journal.pmed.1004049)
Supplement: S1 Table — (DOCX) [file pmed.1004049.s002.docx]

**S1 Table. ICD-10 codes to define infections of interest.**

| **Variable name** | **Codes** | **Diagnosis** |
| --- | --- | --- |
| Skin and soft tissue infections | A48.0 | Gas gangrene |
|  | L02.X | Cutaneous abscess, furnuncle and carbuncle |
|  | L03.X | Cellulitis |
|  | L08.8 | Other specified local infections of skin and subcutaneous tissue |
|  | L08.9 | Local infection of skin and subcutaneous tissue, unspecified |
|  | L97 | Ulcer of lower limb, NEC |
|  | L98.4 | Chronic ulcer of skin, NEC |
|  | L98.8 | Other specified disorders of skin and subcutaneous tissue |
|  | L98.9 | Disorder of skin and subcutaneous tissue, unspecified |
|  | M72.6 | Necrotizing fasciitis |
|  | R02 | Gangrene, NEC |
| Sepsis and bacteraemia | A40.X | Streptococcal sepsis |
|  | A41.X | Other sepsis |
|  | R57.2 | Septic shock |
|  | B37.7 | Candidal sepsis |
| Endocarditis | B37.6 | Candidal endocarditis |
|  | I33.0 | Acute and subacute infective endocarditis |
|  | I33.9 | Acute endocarditis, unspecified |
|  | I34.0 | Mitral (valve) insufficiency |
|  | I34.2 | Nonrheumatic mitral (valve) stenosis |
|  | I34.8 | Other nonrheumatic mitral valve disorders |
|  | I34.9 | Nonrheumatic mitral valve disorder, unspecified |
|  | I35.X | Nonrheumatic aortic valve disorders |
|  | I36.X | Nonrheumatic tricuspid valve disorders |
|  | I37.X | Pulmonary valve disorders |
|  | I38 | Endocarditis, valve unspecified |
|  | I39.X | Endocarditis and heart valve disorders in diseases classified elsewhere |
|  | T82.6 | Infection and inflammatory reaction due to cardiac valve prosthesis |
| Septic arthritis | M00.X | Pyogenic arthritis |
| Osteomyelitis & vertebral discitis | M86.X | Osteomyelitis |
|  | M46.2 | Osteomyelitis of vertebra |
|  | M46.3 | Infection of intervertebral disc (pyogenic) |
|  | M46.4 | Discitis, unspecified |
|  | M89.9 | Disorder of bone, unspecified |
| Central nervous system infections | G06.0 | Intracranial abscess and granuloma |
|  | G06.1 | Intraspinal abscess and granuloma |
|  | G06.2 | Extradural and subdural abscess, unspecified |
