# Supplementary material for: Opioid agonist treatment and risk of death or rehospitalization following injection drug use–associated bacterial and fungal infections: A cohort study in New South Wales, Australia
Source: PLoS Med. 2022 Jul 19;19(7):e1004049. doi: 10.1371/journal.pmed.1004049 (PMC9295981; doi:10.1371/journal.pmed.1004049)
Supplement: S1 Fig — Figure generated with Daggity.net software. Timing of variables generally goes from the left to right. Blue circle is outcome. Green circle is exposure. Red circles are ancestors of exposures and of outcomes. White circles are adjusted variables (in this case, through study design and selection criteria). Gray circles are unobserved variables (in this case, macroenvironmental influences on risk). DAG, directed acyclic graph. (DOCX) [file pmed.1004049.s003.docx]

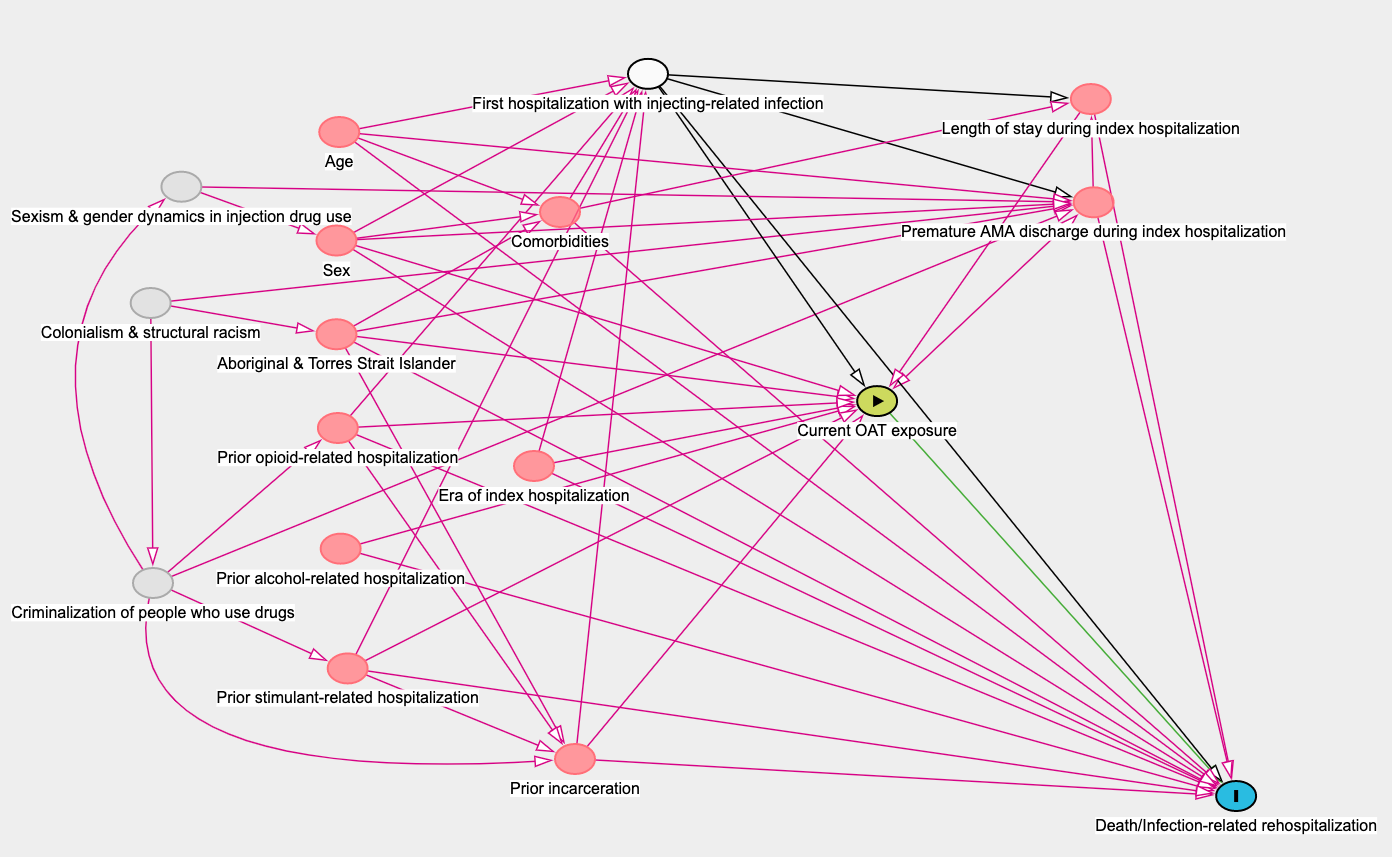


**S1 Fig. Directed Acyclic Graph (DAG) describing hypothesized relationships between primary exposure, covariates, and outcomes.**

Figure generated with Daggity.net software. Timing of variables generally goes from the left to right. Blue circle is outcome. Green circle is exposure. Red circles are ancestors of exposures and of outcomes. White circles are adjusted variables (in this case, through study design and selection criteria). Grey circles are unobserved variables (in this case, macro-environmental influences on risk).
