# Supplementary material for: Opioid agonist treatment and risk of death or rehospitalization following injection drug use–associated bacterial and fungal infections: A cohort study in New South Wales, Australia
Source: PLoS Med. 2022 Jul 19;19(7):e1004049. doi: 10.1371/journal.pmed.1004049 (PMC9295981; doi:10.1371/journal.pmed.1004049)
Supplement: Attachment — Submitted filename: R1_reviewerresponse_OATS_afterhospital_PLOSMed_220502.docx [file pmed.1004049.s004.docx]

Richard Turner, PhD

Senior Editor, *PLOS Medicine*

2022-May-2

**Re: Manuscript Number PMEDICINE-D-22-00493R1**

Dear Prof. Turner,

Thank you for considering our manuscript and for the opportunity to revise and re-submit. We appreciate the reviewers’ close reading of the manuscript and specific, helpful comments. We have attempted to address all comments, below, and we feel they have significantly improved the quality of the manuscript. We have itemized and underlined the changes (line numbers refer to those in the “tracked changes” version of the manuscript, showing “All markup”).

**Requests from the editors:**

**Editor comment 1:** Please add "LD is a member of PLOS Medicine's Editorial Board", or similar, to the competing interest statement (submission form).

**Author response:** Statement added to the competing interest statement in the online submission form: “LD is a member of PLOS Medicine’s Editorial Board”.

**Editor comment 2:** Please review PLOS' data policy (<https://journals.plos.org/plosmedicine/s/data-availability>) and adapt the data statement (submission form) to ensure that authors are not included as contacts for readers interested in inquiring about access to study data.

**Author response:** Change made. The updated statement on the online submission form is now as follows:

- “Requests for data access can be submitted to the National Drug and Alcohol Research Centre (NDARC) at UNSW Sydney (ndarc@unsw.edu.au) and will be reviewed by the OATS investigator team. Approval for the linkage of health data in NSW is provided under strict conditions, to protect confidentiality. Potential collaborators will be required to gain approval for data access and specific secondary analyses from the NSW Population and Health Services Research Ethics Committee. Collaborators proposing to examine research questions relating specifically to Aboriginal peoples will also be required to seek approval from the Aboriginal Health and Medical Research Council. Data may only be analysed within Australia.”

**Editor Comment 2:** We ask you to adapt the title to better match journal style, and suggest: "Opioid agonist treatment and mortality and rehospitalization following injection drug use-associated bacterial and fungal infections: A linkage cohort study".

**Author response:** Change made. As suggested, the title is now, “Opioid agonist treatment and risk of death or rehospitalization following injection drug use-associated bacterial and fungal infections: a linkage cohort study". (We did this to avoid using “and” multiple times, in different ways.)

**Editor comment 3:** Please add a new final sentence to the "Methods and findings" subsection of your abstract, beginning "Study limitations include ..." or similar and quoting 2-3 of the study's main limitations.

**Author response:** Change made to add limitations to abstract “Methods and findings” section:

- Abstract, lines 40-43:

“Study limitations include the use of routinely collected administrative data, which lacks important information on other risk factors for injecting-related infections including injecting practices, injection stimulant use, housing status, and access to harm reduction services (e.g. needle exchange, supervised injecting sites). We also lacked information on OAT medication dosages.”

**Editor comment 4:** After the abstract, please add a new and accessible "Author summary" section in non-identical prose. You may find it helpful to consult one or two recent research papers in PLOS Medicine to get a sense of the preferred style.

**Author response:** Author Summary added:

- Author Summary, lines 48-73:

“**Why was this study done?**

- Injecting-related bacterial and fungal infections are an increasingly common cause of pain, disability, and death among people who inject drugs.
- Treatment of injecting-related infections has tended to focus on antimicrobial therapy and/or surgery, without addressing underlying substance use-related needs.
- Opioid agonist treatment (including methadone and buprenorphine) has been associated with decreased risks of other injecting-related harms (including HIV infection, hepatitis C virus infection, and overdose death), and may be associated with reduced risks of recurrence after injecting-related infections.

**What did the researchers do and find?**

- We identified 8,943 people with opioid use disorder who were admitted to hospital with injecting-related infections in New South Wales, Australia, between 2001 and 2018.
- We found that use of opioid agonist treatment after hospital discharge was associated with both decreased risks of death and of rehospitalization with injecting-related infections.
- Death and rehospitalization with injecting-related infections were common, even among study participants using opioid agonist treatment.

**What do these findings mean?**

- Opioid agonist treatment should be considered as part of a multi-component treatment strategy for injecting-related infections, aiming to reduce death and reinfection.”

**Editor comment 5:** At line 96 and any other instances, please substitute "sex" for "gender" as appropriate.

**Author response:** Change made to consistently use “sex” instead of “gender”, throughout.

**Editor comment 6:** Early in the Methods section (main text), please state whether or not the study had a protocol or prespecified analysis plan, and if so attach the document, referred to in the text.

**Author response:** We did not publish a full protocol before conducting the analyses. Early in the Methods section, we have added the following statements:

- Methods, lines 201-203:

“We did not publish a protocol before conducting the analyses. The main analysis was prespecified before conducting the analyses, but the supplementary and sensitivity analyses were not prespecified.”

**Editor comment 7:** Please highlight analyses that were not prespecified.

**Author response:** In addition to our response to comment 6, above, we have added explicit statements to the new, additional supplementary and sensitivity analyses to highlight that these were not prespecified:

- Methods, Analysis, lines 323-352:

### *“Supplementary analyses*

The relationship between OAT use and the outcomes (mortality or rehospitalization with injecting-related infection) may vary over time, and OAT may have a larger effect closer to the time of initial hospital discharge. As such, we performed a *post hoc* (not prespecified) supplementary analysis to generate period-specific hazard ratios within the first year after hospital discharge, within years 2 to 3, and within years 4 to 6. We did this as an extension of our final multivariable models in the main survival analyses, adjusting for all prespecified covariates.

### *Sensitivity analyses*

We conducted several *post hoc* sensitivity analyses to test the robustness of our main analysis. First, we tested the impact of alternative OAT exposure period definitions. In our main analysis (described above), we prespecified that the six days following the end of an OAT episode is counted as part of the exposure. We tested whether we found similar results when reducing this exposure period to the two days following the OAT episode, and when extending it to 10 days following the OAT episode.

We then conducted a *post hoc* sensitivity analysis to address a potential source of “immortal time bias” in the mortality outcome survival analysis. Immortal time occurs when, within an observation period, there is a period of time where an outcome event cannot possibly have occurred (39,48). Because linkage between OAT record data and hospitalization data was retrospective, some participants may have had their initial hospitalization before their initial OAT record and would have been unable to experience death during this time (in other words, the fact that they have a future OAT record means they could not have died before then). We therefore constructed a new analytic sample only among participants who experienced hospitalization for injecting-related infection after their first record of OAT. We did not feel this potential issue with immortal time bias would affect the rehospitalization outcome survival analysis because participants could have experienced a rehospitalization event at any time (in this case, the fact that they have a future OAT record does not necessarily mean they could not have been hospitalized before then).”

**Editor comment 8:** Throughout the text, please relocate reference call-outs to precede punctuation, e.g., "... similar pathways [1,25].".

**Author response:** Changes made to this formatting throughout.

**Editor comment 9:** Please remove the information on data sharing from the end of the main text. In the event of publication, this information will appear in the article metadata, via entries in the submission form.

**Author response:** Change made. We have removed the data sharing statement from the manuscript.

**Editor comment 10:** In the reference list, please convert all boldface text and italics to plain text.

**Author response:** Change made to reference list formatting.

**Editor comment 11:** Please use the journal name abbreviations "PLoS ONE" and PLoS Med.".

**Author response:** Changes made to reference list formatting.

**Editor comment 12:** Where appropriate, please list 6 author names rather than 3, followed by "et al.".

**Author response:** Changes made to reference list formatting.

**Editor comment 13:** Please add a completed checklist for the most appropriate reporting guideline, e.g., STROBE, as an attachment, labelled "S1_STROBE_Checklist" and referred to as such in the Methods section (main text).

In the checklist, please refer to individual items by section (e.g., "Methods") and paragraph numbers, not by line or page numbers as these generally change in the event of publication.

**Author response:** Checklist completed as instructed and uploaded as supplementary file.

**Comments from academic editor:**

**AE comment 1**: I see that time at risk begins immediately after discharge. Are "bounce backs" (i.e., readmission within a short time interval, e.g. 24 hours, 7 days, etc) therefore considered new hospitalizations? And if so, is there any reason to suspect this may bias the estimated associations away from the null? Perhaps a sensitivity analysis might be reassuring. Either way, please include some discussion of this in the text.

**Author response:** Rehospitalizations could occur at any time (including within a short-interval) but had to be coded as emergency (unplanned) hospitalizations. Our thinking was that if an immediate readmission is an emergency (unplanned) readmission, this would still be a problem that patients, clinicians, and health systems would wish to avoid. We have therefore added the following statements to the manuscript:

- Methods, Measures, Outcomes, lines 259-262:

“Rehospitalizations for injecting-related infections were identified using the same criteria as index hospitalizations, and therefore also had to be coded as emergency (unplanned) admissions. These could occur at any time point in follow-up, so may have included both hospitalizations for new infections or failed treatments of initial infections.”

In response to this comment and Reviewer #3’s request for period-specific hazard ratios, we have added a supplementary analysis for both outcomes exploring hazard ratios within the first year, within years 2 and 3, and within years 4 and 6. Although we have not added a sensitivity analysis merging rapid rehospitalizations with the index hospital admission, the period-specific hazard ratios show that such a sensitivity analysis would be unlikely to show qualitatively different results.

- Methods, Analysis, supplementary analyses, lines 312-319:

“*Supplementary analyses*

The relationship between OAT use and the outcomes (mortality or rehospitalization with injecting-related infection) may vary over time, and OAT may have a larger effect closer to the time of initial hospital discharge. As such, we performed a *post hoc* (not prespecified) supplementary analysis to generate period-specific hazard ratios within the first year after hospital discharge, within years 2 to 3, and within years 4 to 6. We did this as an extension of our final multivariable models in the main survival analyses, adjusting for all prespecified covariates.”

- Results, Other analyses, supplementary analyses, lines 425-433 (and Table 3):

“In a *post hoc* supplementary analysis, we explored associations between OAT and mortality or rehospitalization for injecting-related infections at different points in follow-up using period-specific hazard ratios (Table 3).

Table 3. Period-specific adjusted hazard ratios for associations between opioid agonist treatment and all-cause mortality or rehospitalization for injecting-related infections.

| **Time since hospital discharge** | **Mortality outcome** | **Rehospitalization outcome^1^** |
| --- | --- | --- |
| Within first year | 0.47 (0.40 – 0.55) | 0.83 (0.77 – 0.91) |
| Year 2-3 | 0.66 (0.54 – 0.81) | 0.87 (0.76 – 0.99) |
| Year 4-6 | 0.76 (0.58 – 0.98) | 1.10 (0.91 – 1.33) |

Hazard ratios (with 95% confidence intervals) are for opioid agonist treatment exposure in fully adjusted models for all covariates.

^1^Rehospitalization for injecting-related infection

**AE comment 2:** The authors state that the six days following an OAT episode is counted as part of the exposure. How was the decision made to specify the threshold at six days? Please describe in in the text and/or state whether or not a sensitivity analysis (e.g., with alternative threshold periods) is necessary.

**Author response:** The decision to incorporate six days following the OAT episode into the exposure period was prespecified, based on an approach taken in several prior studies cited in the manuscript. It was initially based on consultation with clinicians and pharmacologists around the duration of action of these medication treatments, and has become somewhat standard in this area of research. We have added further description of his.

However, we acknowledge that this is a convention rather than a specific and reliable reflection of the action of opioid agonist medications. We have therefore added sensitivity analyses changing the definition of the exposure to incorporate two days or 10 days after the end of the OAT episode. These additional sensitivity analyses did not meaningfully change our results:

- Methods, Measures, Primary exposure, lines 266-273:

“Consistent with previous studies, a new OAT episode was defined as one commencing seven or more days after the end date of a prior treatment episode (38–41). The same definition was used for defining the end of OAT episodes, treating the six days following the final day of the prescription as part of the episode. The decision to incorporate the six days following an OAT episode into the exposure definition was originally based on consultation with clinicians and pharmacologists (41); it has been used in previous studies by members of our group (41,42), and similar cut-offs (e.g. three to six days) have been used by others (43,44). This decision may introduce bias by allocating outcomes to the treatment period when they actually occurred after leaving treatment; this may over-estimate rates of outcomes in-treatment (on OAT) and under-estimate rates of outcomes out-of-treatment (off OAT), resulting in conservate estimates of potential benefit.”

- Methods, Analyses, Sensitivity analyses, lines 246-252:

“We conducted several post hoc sensitivity analyses to further explore our findings. First, we tested the impact of alternative OAT exposure period definitions. In our main analysis (described above), we prespecified that the six days following the end of an OAT episode is counted as part of the exposure. We tested whether we found similar results when reducing this exposure period to the two days following the OAT episode, and when extending it to 10 days following the OAT episode.”

- Results, Other analyses, Sensitivity analyses, lines 434-441:

“We conducted *post hoc* sensitivity analyses exploring the impact of alternative OAT exposure timing definitions. Changing our exposure definition to incorporate the two days following the end of the OAT episode (reduced from six days in the main analysis) demonstrated similar results for the association between OAT with all-cause mortality (aHR 0.51, 95% CI 0.46 – 0.57) and with rehospitalization (aHR 0.88, 95% CI 0.83 - 0.95). Extending the exposure period to incorporate 10 days following the end of the OAT episode also demonstrated similar results for mortality (aHR 0.72, 95% CI 0.65 – 0.80) and for rehospitalization (0.89, 95% CI 0.84 – 0.95).”

**AE comment 3:** Relatedly, the authors describe how person-time exposure was counted as "while exposed to OAT" vs. "while unexposed to OAT". Is there any need to account for any lag effects? For instance, suppose a study participant is unexposed to OAT and during this time engages in injection drug use. Then they re-engage in OAT and are therefore counted as exposed. But then 2 days later, due to the injection drug use behavior from several days before, this individual becomes sicker and requires hospitalization. They would be hospitalized but would be counted as exposed.

**Author response:** Thank you for highlighting this point about potential lagging effects in the relationship between OAT exposure and risk of rehospitalization with injecting-related infection. As we describe in the Introduction, the relationship between OAT use and risk of injecting-related infections is complex and may operate through multiple pathways. OAT allows people to avoid injecting, reduce frequency of injecting, or to inject more safely (i.e., if they are less concerned about withdrawal and able to take time and use clean technique). OAT also promotes engagement with health care, e.g. for wounds or treatment of superficial infections before they spread.

We therefore do not believe that one single lag effect would be appropriate to account for these different scenarios. We have added sensitivity analyses changing the duration of time accounted to the exposure (now at two days, six days, and 10 days after OAT episode end) without meaningfully changing our results (see response to comment two, above).

We feel our data and study design support our main conclusions – that use of OAT is associated with modestly reduced likelihood of rehospitalization with injecting-related infections. Our group is planning future analyses of OATS Study data looking at the timing of different exposures and risk of injecting-related infections, e.g. through a self-controlled case series approach.

**AE comment 4:** I would like to see the text provide more speculation about the magnitude of the difference between the effect of exposure on mortality vs. the effect of the exposure on rehospitalization. The large difference, and the fact that the mortality benefit occurs instantaneously (i.e., after discharge), makes me worry about potentially unobserved confounding.

**Author response:** We had added further discussion about the magnitude of the difference between the effects of OAT on the two different outcomes. OAT is known to significantly reduce risks of death even among people who continue to inject opioids, who may still be at risk for injecting-related infections. We propose several pathways through which OAT might reduce risks of death in people

- Discussion, lines 492-504:

In the present study, we identified larger effect estimates for associations between OAT use and mortality than for associations between OAT use and rehospitalization with injecting-related infections. Our findings of a large protective effect of OAT on mortality risk reduction are in keeping with prior research, including multiple observational studies showing protective effects on all-cause mortality, opioid overdose deaths, and multiple other specific causes of death (including suicide, cancer, alcohol-related, and cardiovascular-related) (23,57). Future research should investigate associations between OAT and specific causes of death after hospitalization with injecting-related infections. We hypothesized several pathways through which OAT might reduce risks of recurrence of injecting-related infections, including reducing frequency of opioid injecting, improving health care contacts, and reducing the impacts of criminalization and violence, but we were unable to explore specific mechanisms in this study of administrative data (1,26). People accessing OAT may still be at risks of injecting-related infections through several pathways, including ongoing injection opioid use while on OAT, suboptimal access to safe housing and harm reduction services (e.g. needle exchange, supervised consumptions sites) and by injecting stimulants. OAT is known to reduce risks of death even among people who continue to use nonmedical or criminalized opioids (58), who may still be at risk of injecting-related infections. More research is needed to understand how to further reduce risks of injecting-related infections for people both on and off OAT.”

Regarding the shapes of the extended Kaplan-Meier curves, we expected to see a large mortality risk immediately after discharge as this period is known to be associated with four-times excess risk of death (see Lewer et al. PLOS Med 2021, cited as ref #61 in the manuscript). We therefore also expected the benefit of OAT to be concentrated in this period. The curves also show that the benefit continues throughout follow-up, but the risk difference is smaller because the baseline risk is lower. The extended Kaplan-Meier curves plot risk periods on vs. off treatment, and the time period immediately following OAT is also known to be associated with excess risk (see, e.g. Pearce et al. BMJ 2021, cited as ref #53 in the manuscript). We were looking closely at the shapes of the curves to ensure we did not introduce “immortal time” with our sample or exposure definitions.

**AE comment 5:** Was the effect of exposure similar for Aboriginal or Torres Strait Islanders vs. non-Aboriginal or Torres Strait Islanders?"

**Author response:** We believe this question may be motivated by points made Reviewer #2, whom we think misinterpreted our study design and the hazards ratios presented in Table 2.

We did not investigate potentially differential (or inequitable) effects of OAT among Aboriginal and Torres Strait Islanders vs. non-Indigenous people in New South Wales. Instead, we included Aboriginal and Torres Strait Islander status as a covariate in our multivariable survival analysis of the associations between OAT exposure with mortality and with rehospitalization with injecting-related infections. We interpret our results as suggesting that these associations are meaningful, after adjusting for multiple covariates including Aboriginal and Torres Strait Islander status.

We feel the question of whether the effect of OAT is similar for Aboriginal or Torres Strait Islanders vs. non-Aboriginal participants is beyond the scope of our study (and our research question), and it would be inappropriate to simply stratify the analyses by this covariate.

Access to and retention in OAT among Aboriginal and Torres Strait Islanders have been investigated by others (see, e.g. Bharat et al. 2021 https://doi.org/10.1111/add.15514), and this is a complex issue. The importance of promoting access to culturally safe OAT care for Aboriginal and Torres Strait Islanders is highlighted in OAT prescribing guidance for New South Wales (see, e.g. https://www.health.nsw.gov.au/aod/Publications/nsw-clinical-guidelines-opioid.pdf).

We feel it would be inappropriate to simply stratify our analyses by Aboriginal and Torres Strait Islander status, partly because we have no hypothesis as to why the effect would be different for participants who are “on treatment” and already engaged in OAT. Such interactions can be difficult to investigate and need a clear hypothesis about the cause of the interaction and whether it is expected to be super-additive or super-multiplicative, accounting for differing baseline risks. They also require a lot of power, and given our focus on patients with severe infections we may not identify a real difference in effectiveness. Issues related specifically to preventing injecting-related infections among Aboriginal and Torres Strait Islanders who inject drugs will require dedicated future research, co-led by Aboriginal and Torres Strait Islander elders and community members.

Please also see our response to Reviewer #2, who asked a related question.

**Comments from the reviewers:

*** Reviewer #1:**

I mostly confine my remarks to statistical aspects of this paper. These were generally very well done, but I do have a few comments and suggestions.

**R1 comment 1:** p. 2 line 32   What is the number after the ± sign?  An sd? A CI? Something else? Please specify.

**Author response:** We have edited the abstract to clarify that this is a standard deviation associated with mean age:

- Abstract, Methods and Findings, line 34:

“The study included 8,943 participants (mean age 39, standard deviation 11 years; 34% women).”

**R1 comment 2:** p. 4 line 48-49  "Many parts of the world" ought to include some places in Africa, Asia, and less developed countries generally. If there's no data on those countries then maybe say "in many developed countries" or something like that.

**Author response:** We have changed the text to include additional references to increasing incidence of hospitalizations for injecting-related bacterial infections in South Africa and India:

- Introduction, lines 93-95:

“The incidence of hospitalization for injecting-related infections is increasing in many parts of the world, including Australia (7), Canada (2,8,9), South Africa (10), the United Kingdom(11), the USA (12–16), and India (17)**.**

**R1 comment 3:** p. 6-7 line 104-5   Maybe I am missing something but are the "eligible index hospitalizations" the control group?  The linking mechanism is described earlier, but then this group doesn't seem to be described in the section on particiapants.

**Author response:** We apologize we were inconsistent with terminology here, which likely contributed to confusion. “Eligible index hospitalizations” was simply meant to refer to the final selection of included participants, who needed to have an unplanned (emergency) hospital admission for an injecting-related infection that ended in discharge, alive, to the community. This was so that the timing of potential exposures and potential outcomes were aligned, in order to avoid problems with “immortal time”. We have added that explicit explanation in the text:

- Methods, Participants, lines 232-237:

“To be eligible, these hospitalizations had to end with the participant discharged alive to the community (rather than transfer to another hospital or care facility) so that participants could be eligible for OAT outside the hospital (see Figure 1). This was so that the timing of potential exposure and potential outcome were aligned, to avoid problems with “immortal time bias” when participants would be unable to experience either the exposure (OAT outside of acute care hospitals) or the outcomes (rehospitalization or death) (34).”

**R1 comment 4:** p. 8 line 139  Centering age is fine, but I am not a fan of scaling variables to 1 SD. I know some statisticians do recommend it, but I think it makes things a little less clear.  For age (as here) a year is a year, but an SD in one study is different from an SD in another study. I won't insist on changing this, but I do recommend using age. (Same comment for LOS, below).

**Author response:** Thank you for this point. We understand there are different approaches to the scaling of covariates in multiple regression models and we will consider additional, unscaled approaches in future analyses.

**R1 comment 5:** p, 9 line 148-9  Why group admission? Categorizing continuous variables is not recommended. It increases both type 1 and type 2 error and introduces a kind of "magical thinking" that something big happens at the cutpoints. Instead, leave year as a number and use splines to investigate nonlinearity.  (Splines could also be used with age).

**Author response:** We interpret this comment as asking specifically why we treated hospitalization date as groups of years (i.e. 2001-2006, 2007-2011, or 2012-2018) rather than leaving as a continuous variable. In brief, as described in our DAG, we are interested in year of hospitalization only as a covariate and potential confounder, to try to account for potential secular trends in risks of death or rehospitalization over time. We felt that grouping the years in this way would allow for readers to get a general sense of the distribution of dates (in Table 1) while also accounting for possible secular trends in multivariable regressions. We do not further investigate outcomes or incidence of infections over time, but this could be done in the future with different study designs.

In response to this comment, we have conducted some further analyses with different treatments of the date variable. When we treat hospitalization date as a continuous integer by day or group it by calendar year (rather than groups of multiple calendar years), this does not change our adjusted hazard ratio for our primary exposures of OAT in the multivariable survival analyses. The conditional hazard ratios for hospitalization date in the fully adjusted mortality survival analysis are 1.00 (0.99-1.00) when treated as continuous by day and 0.98 (0.97-0.99) when treated as continuous by calendar year.

We therefore decided to report the results by groupings of year, as this was our prespecified analysis plan.

**R1 Comment 5:** Table 1 - LOS is clearly very skew. Giving median and IQR is fine, but a density plot of this variable would give more insight. What accounts for the huge SD? Is it a single outlier (someone with 500 days or something) or is it more general right skew? A density plot or quantile plot would reveal this.

**Author response:** Length of stay is indeed very right-skewed, and this is usually the case in hospital data. While the median is four days and the 75^th^ percentile is eight days, the 99^th^ percentile is 65 days. We have added this description to the text:

- Results, participants, lines 278:

“Length of stay was right skewed, with median four days, 75^th^ percentile eight days, and 99^th^ percentile 65 days.”

Removing length of stay from multivariable models does not affect our results.

Peter Flom

***** Reviewer #2:**

In this study, Brothers et all use data from the Australia OAT registry to example how the exposure of recent OAT relates to 2 outcomes: death and hospitalization.  The authors are clearly very informed about the subject area, and I know their work well. My major issue with this is that the authors do not highlight as much why this specific registry is interesting and notable. There is a tension between "We know this" and "We don't know this." After reading this introduction, it is a hard sell to me that this work needs to be done. But, as you read the methods more, you realize how special this data set is because it is a closed system with really granular data. I say closed system not exactly knowing how many people leave New South Wales to go live in other places in Australia. Overall I think it is less than, say, people moving from Massachusetts to Rhode Island or New Hampshire. We would never be able to publish this type of data in the US.

**R2 comment 1:** Additionally, 15% of people in this study are aboriginal. And I am not sure how comfortable the authors feel about diving into the details about disparities in OAT to aboriginal people, but this seems like the right time to use this data to make that statement. Yes, it will not work towards the idea of "generalizability" because not all readers will interact with aboriginal people. But it is hard to find this type of database with high percentages of native americans, and it could at least be the start to a conversation about why certain people are left out of the conversation.  It looks like in the adjusted analysis the benfit of OAT goes away for both outcomes. So, I think this deserves to be published but the authors need to work on convincing me to read this paper. And the angle to sell this paper is the unique data set and the health disparities angle for sure. I recommend reframing the paper. Benefits of OAT in PWID Not Seen In Aboriginal People: Why?

**Author response:** We appreciate the reviewer raising this issue, as the health of Indigenous people in settler-colonial countries (whether Aboriginal and Torres Strait Islanders in Australia, First Nations people in Canada, or Native Americans in the US) is a very important topic for research – particularly as it relates to the effects of settler-colonialism and structural racism on substance use-related outcomes.

In our study, however, we did not look at whether the effects (or associations) of OAT exposure on mortality or rehospitalization differ by Aboriginal status. We believe the reviewer may have misinterpreted the Hazard Ratios (HRs) presented in Table 2. The unadjusted and adjusted HRs presented in Table 2 in each row refer to estimates for the measured associations between that covariate (ie., Aboriginal or Torres Strait Islander status) and each outcome (ie., mortality or rehospitalization with injecting-related infections). This row in Table 2 shows that Aboriginal and Torres Strait Islander status is associated with a lower hazard of mortality in univariate analyses (unadjusted HR 0.72, 0.61-0.85), but fully adjusted analyses found an estimate that could include both increased and decreased risk (adjusted HR 1.02, 0.86-1.20). For the rehospitalization outcome, associations with Aboriginal and Torres Strait Islander status are nonsignificant and relatively closer to a null association in both unadjusted (HR 0.95, 0.86-1.04) and fully adjusted (aHR 1.00, 0.91-1.10) analyses.

To help ease understanding, we have added the reference category for all variables in Table 2.

Also please see response to your comment 13, below.

**R2 comment 2:** *(Introduction)* Concise and overall well done. In the first paragraph I might say specifically what the primary and secondary prevention methods are (safe injection sites, early identification of OUD, decreased barriers to OAT access). On the revisions it makes sense to reframe talking about major issues with known data that this paper/this data set can help answer. If you look at work by leaders like Laura Marks or Sim Kimmel, I think in the have people who are "lost to follow up" or they discuss issues with the linkage of data. I believe it has been part of my work as well, because I can only tell if people die in MA. I also don't think you can say OAT has not been prioritized without saying there are some places who have prioritized OAT, but many places have not.  Do you think there is limited evidence that OAT prevents against death and rehospitalization?  I think that is a stretch. I think there is more and more. And maybe that is where my disconnect lies with the paper. I think there is a lot of evidence about OAT protecting against hospitalization and death. I like your comment about limited sample size. It gives you another avenue to say this is a large group of people. Also please highlight more that this cohort includes incarcerated people (or, is it history of incarceration?). That is an important point and distinction.

**Author response:** Thank you for encouraging us to expand on the rationale for this study and the value of our dataset and study design. We had kept the Introduction brief and focused as a stylistic choice, but we have now expanded it greatly in an effort to respond to all the points in this comment.

While there is indeed robust prior observational evidence showing an association between OAT use and reduced mortality among people with opioid use disorder, the prior evidence regarding prevention of injecting-related infections is far more limited and far less clear:

- Introduction, lines 77-183:

“Prevention efforts to date have focused on individual-level behaviour change interventions to promote cleaner drug preparation and safer drug injecting technique, but these have shown mixed results (18–20) and have had limited impact on a population level (1). This may be in part because of social and structural factors (e.g. criminalization, discrimination, lack of access to housing, harm reduction services, and supervised injection sites) that constrain the ability of PWID to inject more safely (1,21) and that push PWID away from health care (22). Improved primary and secondary prevention approaches are urgently needed (1,13,22).

One promising potential intervention to prevent injecting-related bacterial and fungal infections is opioid agonist treatment (OAT; e.g. methadone or buprenorphine). For people with opioid use disorder, OAT is associated with many benefits including reduced risks of death and of viral infections including human immunodeficiency virus (HIV) and hepatitis C virus (23,24). OAT limits opioid withdrawal symptoms, reduces reliance on illicit drug markets, and empowers PWID to inject less frequently or in a safer way (25,26). Engagement in OAT is also associated with regular health care contacts where superficial infections may be treated before they progress and become more severe or spread through the bloodstream (22,27,28).

Despite these possible benefits, in many acute care hospitals OAT is not prioritized as part of treatment planning during and after hospitalization with injecting-related bacterial and fungal infections (22,29–31). This is represented in low rates of OAT prescribing for these patients in multiple studies from North America (29,31,32) and in qualitative studies from the United Kingdom (1). Suboptimal access to OAT may reflect system-level issues which separate addiction care from specialized, acute medical care for infections (1,22,29,30). In some hospitals, clinicians have tried to overcome this by establishing specialized addiction medicine consultation services (1–4) or by infectious diseases specialists prescribing OAT directly (1,2). While OAT is known to be beneficial for other injecting-related health outcomes, there has been relatively little research on OAT and risk for injecting-related infections. A better understanding of how OAT affects outcomes after injecting-related infections could help inform treatment planning during and following hospitalization.

Prior analyses of potential benefits of OAT after hospitalization with injecting-related infections have been limited by small sample sizes with wide confidence intervals (38,39). Three administrative linkage cohort studies (all from U.S. insurance claims data) have assessed associations between use of OAT and outcomes after hospitalization with injecting-related bacterial or fungal infections (39–41). One study identified a reduced risk of death after hospitalizations with injecting-related endocarditis, but did not assess rehospitalizations (40). A second study identified no significant effect (with wide confidence intervals) on risk of rehospitalization after endocarditis and did not assess mortality (39). A third identified a reduced risk of rehospitalization for skin and soft-tissue infections at one year (41). Reflecting suboptimal access, use of OAT (or of naltrexone, an opioid antagonist medication used for opioid use disorder treatment in the U.S.) was reported as 24% within 3 months following hospital discharge in the first study (40) and as 6% within 30 days following discharge in the second and third studies (39,41). The latter two studies also only included information on buprenorphine use, as they did not have access to insurance claims or prescribing records for methadone.

The Opioid Agonist Treatment Safety (OATS) Study is an administrative data linkage cohort study in New South Wales, Australia, that includes OAT permit records (with methadone or buprenorphine) for every person accessing OAT for opioid use disorder treatment in New South Wales from 2001 to 2018 (1,2).

Using data from the OATS Study, we aimed to evaluate whether OAT after hospitalization with injecting-related bacterial and fungal infections is associated with decreased risk of subsequent mortality or infection-related rehospitalization.”

In response to your comment 12, below, we have further clarified that this dataset includes linkage to incarceration records and specified how this was defined in our study:

- Methods, Setting and data sources, lines 200-202:

“All individuals with an OAT permit were linked to state-wide hospitalization records, incarceration records, and vital statistics/death records between August 2001 and August 2018 using probabilistic linkage based on names, sex, date of birth and Indigenous status (36).”

- Methods, Measures, Covariates, line 283-284:

“All covariates were extracted from linked hospital administrative records, unless otherwise specified.”

- Methods, Measures, Covariates, lines 293-295:

“Participant characteristics measured prior to the index hospitalization (all treated as binary) include any prior acute care hospitalizations related to poisoning or toxicity from opioids (as indicators of addiction severity; T40.0 – T40.6), alcohol (F10.0, X45, X65, Y15, T51.0), or stimulants (T40.5 T43.6), and a history of prior incarceration (which is associated with increased risk for unsafe injection practices). Dates of incarceration were derived from linked incarceration administrative records.”

**R2 comment 3:** *(Methods)* What is an unplanned hospitalization? You mean if they were delivering a baby, and happened to note there was cellulitis, they did not get included? If they had a planned hospitalization and found to have some sort of infection, why would they not be included? Maybe there is a better word for "unplanned" that can help me understand? I am curious about the 776 planned hospitalizations. I am guessing they were planned AND there was no infection?

**Author response:** Thank you for catching this and for encouraging us to clarify. We have changed the language to “emergency (unplanned)” and added further details on these unplanned hospitalizations. This was prespecified to determine eligibility for both inclusion in the analytic sample (participants who survived an emergency hospitalization for injecting-related infections), and to indicate the rehospitalization outcome (these rehospitalizations had to be coded as emergency / unplanned). We felt emergency (unplanned) hospitalizations for acute injecting-relating infections capture the patient population of interest, and that planned hospitalizations (including those when an injecting-related infection was present) would be more likely to represent planned follow-up care and diagnostic imaging/procedures. The coding of hospitalizations as emergency (unplanned) or routine/planned comes from the New South Wales hospital administrative records. In the OATS study (as previously reported), the most common reason for a planned admission was dialysis (18% of planned admissions) (see Lewer et al. DAD 2020, https://doi.org/10.1016/j.drugalcdep.2020.108343).

- Methods, Participants, line 205:

“We included OATS study participants who survived at least one emergency (unplanned) hospitalization with skin and soft-tissue infection, sepsis or bacteraemia, endocarditis…”

- Methods, Participants, lines 219-224:

“Eligible hospitalizations had to be emergency (unplanned) admissions. We excluded routine or planned admissions (e.g. for physical therapy or diagnostic procedures) because they are unlikely to represent episodes of acute illness attributable to injecting-related infections.”

**R2 comment 4:** *(Methods)* I understand why you are selecting only outpatient OAT, but I think you need to be clearer in the intro about this. Also, if someone is going to a rehab on OAT, I would think they are sicker and more likely to be rehospitalizated. That is 1/10th of your sample (983 people). Is it possible to look at rehospitalization and death in the 983 people who did not go to community? Can you include this in your conclusions? You are selecting a healthier population over all.

**Author response:** Thank you for inviting us to clarify this. We did indeed measure use of OAT after discharge from hospital, as a time-varying exposure. We did not have access to hospital prescribing records, but instead used the dates of OAT access from state-wide prescribing permits (as described in the Methods, Setting and data sources section). While this was “outpatient” in the sense that patients had been discharged from their acute care hospitalization, an unknown minority of patients may have attended residential addiction treatment at some point during their follow-up. Patients enrolled in residential addiction treatment would still require a state government OAT permit, which would be captured in our study. Outpatient OAT is recommended as first line treatment for opioid use disorder in Australian and international guidelines (including U.S. and Canadian), and residential treatment without OAT is specifically recommended against as it leads to loss of opioid tolerance and increases risks of overdose death. Use of OAT was our exposure of interest (regardless of outpatient or residential treatment setting).

We have described our exposure variable more clearly in the Methods:

- Methods, Primary exposure, lines 249-252:

“The primary exposure was use of OAT defined by dates with an active OAT prescription. OAT exposure was treated as time-varying, by day of receipt. This means that each participant’s follow-up time was divided into exposed (on OAT) and unexposed (off OAT) periods (ie, medication status was not necessarily constant through follow-up).”

Regarding discharge to the community, we included in our sample at baseline only participants with a hospitalization for injecting-related infection that ended with the participant discharged alive to the community (and not discharged to another acute care facility, e.g. for physical rehabilitation). Patient who were not discharged to the community (and were selected out at that stage) were typically transferred to another hospital for continued acute treatment and on discharge would be eligible for our study. This was prespecified, and we did it so that the timing of our potential exposure (OAT outside of acute care hospitals) was aligned with potential outcomes (rehospitalization or mortality) and to avoid immortal time where neither could be experienced. For participants that were transferred to another hospital (including physical rehabilitation hospitals), they would have been eligible to be included in the sample if they met eligibility criteria at any point during that (or a subsequent) hospitalization. Discharge to a residential addiction treatment facility, which would be rare in our sample, would not lead to exclusion from the study (as this was still discharge out of the acute care hospital). We have added this explanation including a reference describing problems of immortal time with other study designs:

- Methods, Participants, lines 227-235:

“To be eligible, these hospitalizations had to end with the participant discharged alive to the community (rather than transfer to another hospital) so that participants could be eligible for OAT outside the hospital (see Figure 1). This was so that the timing of potential exposure and potential outcome were aligned, to avoid problems with “immortal time bias” when participants would be unable to experience either the exposure (OAT outside of acute care hospitals) or the outcomes (rehospitalization or death) (39).”

**R2 comment 5:** *(Methods)* mycobaterial infections excluded? We have peopel with injection related mycobacterium.

**Author response:** Mycobacterial infections were not necessarily excluded. In keeping with prior studies, diagnostic categories were defined primarily by clinical syndrome (e.g. skin and soft-tissue infection, endocarditis, etc.) rather than specific pathogen. If the mycobacterium was associated with an infection of skin, soft tissue, bones, joints, blood, or heart (which we believe is what you are suggesting) it would be captured by these ICD codes. We did not include pulmonary tuberculosis or non-tuberculous mycobacterial lung infections as we did not conceptualize these as typically introduced through the drug injecting process.

**R2 comment 6:** *(Methods)* Was the primary exposure before hospitalization, during hospitalization, or could be after hospitalization? Did they meet the primary exposure if they got just 1 day of meds? --> (notes from later. Ok, I see in the table it is y/n prescription active at discharge; why do you need time varying based on day of receipt? not sure what that means.)

**Author response:** Thank you for the invitation to clarify this point, which is very important for readers to understand clearly. The primary exposure was use of OAT following participants’ hospitalization with an injecting-related infection. This was treated as time-varying, so that both exposed (on OAT) and unexposed (off OAT) time was included in the model. We have clarified this further in the Methods section:

- Methods, Primary Exposure, lines 249-252:

“The primary exposure was use of OAT, defined by dates with an active OAT prescription. OAT exposure was treated as time-varying, by day of receipt. This means that each participant’s follow-up time was divided into exposed (on OAT) and unexposed (off OAT) episodes (ie, medication status was not necessarily constant through follow-up) (1).

The information on “yes/no” prescription active at discharge was simply used to describe the proportion of the sample starting in an exposed (i.e., receiving OAT) time period and give an indication of potentially unmet care needs. That binary “yes/no” exposure was not used in the survival analysis.

**R2 comment 7:** *(Methods)* Is it possible to go one step back from the 40,000 people and let me know how many people were in the OATs cohort all together on Figure 1

**Author response:** The number of total possible OATS Study cohort members is 48,148. We have added this to Figure 1. We cite the full Data Resource Profile (Larney et al. IJE 2020 https://doi.org/10.1093/ije/dyaa125) describing the full study sample.

**R2 comment 8:** *(Methods)* History of incarceration may be linked to high risk injection practices, but I think current incarceration less so. I just think you need to justify/clarify this statement more.

**Author response:** While we treated the primary exposure of OAT as time-varying, we treated all covariates as fixed (not time-varying) based on status at baseline. This means that we only included information on a history of past incarceration. We added further clarification and we also added this to the Limitations.

- Methods, Measures, Covariates, lines 293-295:

“Participant characteristics measured prior to the index hospitalization (all treated as binary) include any prior acute care hospitalizations related to poisoning or toxicity from opioids (as indicators of addiction severity; T40.0 – T40.6), alcohol (F10.0, X45, X65, Y15, T51.0), or stimulants (T40.5 T43.6), and a history of prior incarceration (which is associated with increased risk for unsafe injection practices). Dates of incarceration were derived from linked incarceration administrative records.”

- Discussion, limitations, lines 519-521:

“These covariates were treated as time-fixed at baseline (i.e., not time-varying); further research is needed to understand whether social exposures like incarceration have time-dependent effects on injecting-related infections.”

**R2 comment 9:** *(Methods)* I might be confused because I don't know Poisson well, but if Mr Jones is on and off OAT, does the days he is on OAT qualify for one of the exposures and the days he is off qualify him for the other? Or does he just fall into one bucket.

**Author response:** The OAT exposure category was time-varying, which meant that exposure status was not necessarily constant (i.e., “one bucket”) throughout follow-up. We have added further explanation to clarify this:

- Methods, Measures, Primary Exposure, lines 257-260:

“The primary exposure was use of OAT defined by dates with an active OAT prescription. OAT exposure was treated as time-varying, by day of receipt. This means that each participant’s follow-up time was divided into exposed (on OAT) and unexposed (off OAT) episodes (ie, medication status was not necessarily constant through follow-up) (1).”

**R2 comment 10:** *(Results)* I find the distribution of the infections fascinating. Such a high amount of hospitalizations for skin and soft tissue infections. Would be great if you could add somehting letting me know skin and soft tissue + something else.

**Author response:** Skin and soft-tissue infections are indeed much more common than other types of deeper injecting-related infections. We apologize we don’t understand the remainder of the comment.

**R2 comment 11:** *(Results)* lines 174 further confuses me. Is it TIME of index hospitalization? Or DISCHARGE?  I need more clarity of the exposure of interest. I think I am confused becayse you are using it in 2 ways. Y/N, and then how much exposure for the KM curve.

**Author response:** Line 174 (now line 348) describes the proportion of participants with an active OAT permit at the time of discharge from their index hospitalization (i.e., their first time-varying exposure period during follow-up began “on OAT”). We have added further description to help aid understanding of the time-varying exposure definitions; also see response to comments 6 and 9:

- Methods, Measures, Primary Exposure, lines 257-260:

“The primary exposure was use of OAT, defined by dates with an active OAT prescription. OAT exposure was treated as time-varying, by day of receipt. This means that each participant’s follow-up time was divided into exposed (on OAT) and unexposed (off OAT) episodes (ie, medication status was not necessarily constant through follow-up) (1).”

**R2 comment 12:** *(Results)* Only 43% had prior experience of incarceration? How did you collect this data? Can you add into methods? Self report or pulled from admin data?

**Author response:** This was from linked incarceration administrative records. We have added text to clarify this:

- Methods, Setting and data sources, lines 211-214:

“All individuals with an OAT permit were linked to state-wide hospitalization records, incarceration records, and vital statistics/death records between August 2001 and August 2018 using probabilistic linkage based on names, sex, date of birth and Indigenous status, as described in the OATS study protocol (36).”

- Methods, Measures, Covariates, lines 293-295:

“Participant characteristics measured prior to the index hospitalization (all treated as binary) include any prior acute care hospitalizations related to poisoning or toxicity from opioids (as indicators of addiction severity; T40.0 – T40.6), alcohol (F10.0, X45, X65, Y15, T51.0), or stimulants (T40.5 T43.6), and a history of prior incarceration (which is associated with increased risk for unsafe injection practices). Dates of incarceration were derived from linked incarceration administrative records.”

**R2 comment 13:** *(Results)* What do. you think about changing your KM curves into stratified analysis of Aboriginal and note. The Aboriginal peopel are not seeing the benefit. I feel like that is the biggest statemennt you can make. There is benefit but it is not equal.

**Author response:** We did not investigate potentially differential (or inequitable) effects of OAT among Aboriginal and Torres Strait Islanders vs. non-Indigenous people in New South Wales, and we feel that question is beyond the scope of our study (and our research question). Access to and retention in OAT among Aboriginal and Torres Strait Islanders have been investigated by others (see, e.g. Bharat et al. 2021 https://doi.org/10.1111/add.15514), and this is a complex issue. The importance of promoting access to culturally safe OAT care for Aboriginal and Torres Strait Islanders is highlighted in OAT prescribing guidance for New South Wales (see, e.g. https://www.health.nsw.gov.au/aod/Publications/nsw-clinical-guidelines-opioid.pdf).

We feel it would inappropriate to simply stratify our analyses by Aboriginal and Torres Strait Islander status, partly because we have no hypothesis as to why the effect would be different for participants “on OAT” vs. “off OAT”. If the effects are different, our data could not tell us how to interpret this within our current study design. Issues related specifically to preventing injecting-related infections among Aboriginal and Torres Strait Islanders who inject drugs will require dedicated research, preferably co-led by Aboriginal and Torres Strait Islander elders and community members.

Your statement that “The Aboriginal people are not seeing the benefit” may reflect a misunderstanding of the hazards ratios presented in Table 2, as we described in response to your comment 1, above. To reiterate, we did not find (nor did we investigate) differential effects of OAT by participants’ Aboriginal status. We do think this is a very important issue, and worthy of future study.

**R2 comment 14:** *(Discussion)* Rework discussion to be around equity. You found something, but it was not equitable benefit. That I believe is how this paper really makes changes and advances the discussion

**Author response:** See response to comment 1 and comment 13. We did not investigate, nor did we find, differential or inequitable effects of OAT among different subgroups. We believe this comment may also be motivated by a misunderstanding of the hazard ratios presented in Table 2. We hope the manuscript is now clearer, and you understand why we cannot rework the Discussion around this issue.

**R2 comment 15:** *(Discussion)* LIne 285-288. Not super relevant to your discussion. You excluded these people

**Author response:** Lines 285-288 refer to the importance of people with opioid use disorder engaging in OAT as soon as possible during or after hospitalization. We did not exclude these people. We apologize if there has been some kind of misunderstanding here as well.

**R2 comment 16:** *(Discussion)*Discuss the other limitations, like excluding people who go to rehab from your analysis.

**Author response:** We excluded people who were discharged from one acute care hospital to another hospital facility (e.g. a subacute physical rehabilitation facility). In case you are referring to addiction “rehab” (residential treatment) facilitates, we did not have information on time spent in inpatient addiction treatment facilities. However, all patients receiving OAT (no matter their residence) would need to have an active permit with the New South Wales state government, and so would be included in our analysis.

**R2 comment 17:** *(Discussion)* Limiations of how places without high percentages of aboriginal people may not feel this applies, but it is about "MINORITIZED" communities overall, not necessarily WHY they are minoritized

**Author response:** We apologize we may not fully understand this comment. Our study was conducted in a specific setting (New South Wales, Australia) which differs from other settings in many ways (including factors related to health care access, financing, insurance, health workforce education, solubility of the heroin supply, etc.) in addition to differences in distributions of population demographics. We don’t feel we can make any general inference about “minoritized” communities overall based on this data.

***** Reviewer #3:**

This manuscript describes the association of opioid agonist treatment (methadone and buprenorphine) with mortality and re-hospitalization following injection drug use-associated bacterial and fungal infections in New South Wales, Australia between 2001 and 2018. The authors find that current OAT use is associated with both decreased rehospitalization and mortality in adjusted models. Hospitals, public health practitioners, infectious disease and addiction clinicians all seek to improve care for people with injection-related infections and this manuscript adds to the evidence. The manuscript is clear and well written and makes an important contribution to the field. I have several suggestions however for the authors to consider which I believe would improve the strength of this manuscript.

**R3 comment 1:** It seems that Individuals in the OATS cohort who experienced a designated hospitalization for infection were eligible for inclusion regardless of whether OAT was received prior to the hospitalization or after the hospitalization. Though this may be a small number of individuals, it is possible that someone could have received OAT only several years after experiencing a designated hospitalization and still be included in the cohort. This represents a form of immortalized time bias --- individuals had to have survived the period after the hospitalization in order to be included in the cohort. This potential bias however would be toward the null. To protect against biased estimates of effect, the authors could consider only including individuals with any OAT receipt prior to the hospitalization in the cohort.

**Author response:** Thank you for your close reading of our manuscript and for this thoughtful comment. We agree that this would be a potential source of immortal time bias for the mortality outcome survival analysis (i.e., the fact that these individuals do have an OAT record in the future means that they could not have died between that hospital discharge and their first OAT record). We have added a sensitivity analysis where we recreate the analytic sample, selecting only from the subgroup of OATS study participants who had an OAT record before an injecting-related hospitalization record. As we now describe in the manuscript, this did not meaningfully alter our conclusions:

- Methods, Analysis, Sensitivity analyses, lines 318-332:

“We then conducted a sensitivity analysis to address a potential source of “immortal time bias” in the mortality outcome survival analysis. Immortal time occurs when, within an observation period, there is a period of time where an outcome event cannot possibly have occurred (39,48). Because linkage between OAT record data and hospitalization data was retrospective, some participants may have had their initial hospitalization before their initial OAT record and would have been unable to experience death during this time (in other words, the fact that they have a future OAT record means they could not have died before then). We therefore constructed a new analytic sample only among participants who experienced hospitalization for injecting-related infection at some date after their first record of OAT. We did not feel this potential issue with immortal time bias would affect the rehospitalization outcome survival analysis because participants could have experienced a rehospitalization event at any time (in this case, the fact that they have a future OAT record does not necessarily mean they could not have been hospitalized before then).”

- Results, sensitivity analyses, lines 421-427:

“We then conducted a *post hoc* sensitivity analysis for the mortality outcome, reconstructing the analytic sample only among participants who experienced hospitalization for injecting-related infection at a date following their first record of OAT. This sample was slightly smaller (n=7,641). Compared to the main analysis, more participants (59%) had an active OAT permit at the time of discharge from their index hospitalization and more follow-up time was exposed to OAT (59%). In the fully adjusted model in this smaller sample, OAT was also associated with reduced hazard of all-cause death (aHR 0.56, 95% CI 0.51 – 0.62).”

**R3 comment 2:** Figure 2 would be more accurate if it also designated OAT receipt.

**Author response:** We included OAT receipt in Figure 2 in the shaded “Exposure window” box.

**R3 comment 3:** The study includes differential follow up time after the hospitalization. The analysis includes methods to address this but assumes that the impact of OAT is the same throughout the entire follow up period. Given that the relationship to the infection and the outcome varies over time (ie. infection may be less relevant to the outcomes years out), it would be instructive to report time varying hazards for example (ie. year 1, year 2-3, and year 4-6).

**Author response:** Thank you for this suggestion. Based on this suggestion, we have added “piecewise” hazard ratios up to year 1, year 2-3, and year 4-6. To do this, we used the “survSplit” function in the “survival” package in R:

- Methods, analysis, supplementary analysis, lines 310-317:

### **“*Supplementary analyses***

The relationship between OAT use and the outcomes (mortality or rehospitalization with injecting-related infection) may vary over time, and OAT may have a larger effect closer to the time of initial hospital discharge. As such, we performed a *post hoc* (not prespecified) supplementary analysis to generate period-specific hazard ratios within the first year after hospital discharge, within years 2 to 3, and within years 4 to 6. We did this as an extension of our final multivariable models in the main survival analyses, adjusting for all prespecified covariates.”

- Results, supplementary analysis, lines 421-430:

“**Supplementary analyses**

In a *post hoc* supplementary analysis, we assessed whether associations between OAT and outcomes changed over time since hospital discharge. As shown in Table 3, hazard ratios were generally lower sooner in follow-up.

Table 3. Period-specific adjusted hazard ratios for associations between opioid agonist treatment and all-cause mortality or rehospitalization for injecting-related infections.

| **Time since hospital discharge** | **Mortality outcome** | **Rehospitalization outcome^1^** |
| --- | --- | --- |
| Within first year | 0.47 (0.40 – 0.55) | 0.83 (0.77 – 0.91) |
| Year 2-3 | 0.66 (0.54 – 0.81) | 0.87 (0.76 – 0.99) |
| Year 4-6 | 0.76 (0.58 – 0.98) | 1.10 (0.91 – 1.33) |

Hazard ratios (with 95% confidence intervals) are for opioid agonist treatment exposure in fully adjusted models for all covariates.

^1^Rehospitalization for injecting-related infection”

**R3 comment 4:**. Figure 3 would be improved with life tables. Also, the average follow up time is 6 years, but the figure only include 3 years of follow up?

**Author response:** We have added a “number at risk” table to the bottom of figure 3 for both panels, and also extended the X-axis on the figure to longer duration of follow-up. Note that in this approach to extended Kaplan-Meier plots, we are plotting the treatment periods rather than the individual participants. (See new Figure 3.)

**R3 comment 5:** This may be beyond the scope of this study, but given the limited evidence in the literature, it may be instructive to also report how the association between OAT and mortality and rehospitalization varies by type of infection (e.g. skin and soft tissue vs more serious infections). Such an analysis would be instructive but could also represent its own manuscript in the future.

**Author response:** Thank you for this suggestion. We conceptualize all these infections as sharing the same pathophysiology (i.e., vascular damage and pathogenic bacteria introduced into sterile compartments through unsafe injection drug use, with or without hematogenous spread). In this “as treated” analysis, we have no hypothesis as to why associations between OAT and the study outcomes would differ by infection type. We agree it would be interesting to explore this data further in a future study and see if access to OAT or patterns of OAT adherence, etc., differ by infection type.

We are grateful for your consideration of this paper and feel the reviewers’ suggestions have improved it significantly. We look forward to hearing from you regarding the disposition of the manuscript.

Sincerely, on behalf of all authors,

**Thomas D. Brothers, MD CISAM**

Canadian Institutes of Health Research (CIHR) Fellow

UCL Collaborative Centre for Inclusion Health

University College London

Visiting Fellow

National Drug and Alcohol Research Centre (NDARC)
UNSW – Sydney

Resident Physician (General Internal Medicine & Clinician-Investigator Program)

Department of Medicine
Dalhousie University
