# Supplementary material for: Opioid agonist treatment and risk of death or rehospitalization following injection drug use–associated bacterial and fungal infections: A cohort study in New South Wales, Australia
Source: PLoS Med. 2022 Jul 19;19(7):e1004049. doi: 10.1371/journal.pmed.1004049 (PMC9295981; doi:10.1371/journal.pmed.1004049)
Supplement: Attachment — Submitted filename: R2_response_OATS_afterhospital_PLOSMed_220609.docx [file pmed.1004049.s005.docx]

Richard Turner, PhD

Senior Editor, *PLOS Medicine*

2022-June-9

**Re: Manuscript Number PMEDICINE-D-22-00493R2**

Dear Prof. Turner,

Thank you for considering our manuscript and for the opportunity to revise and re-submit. Below we address the outstanding requests from editors We have itemized and underlined the changes (line numbers refer to those in the “tracked changes” version of the manuscript, showing “All markup”).

**Requests from the editors:**

**Editor comment 1:** We ask you to remove or reword "... will be reviewed by the OATS investigator team" from the data statement. This seems to suggest that the author group can prevent data release to interested parties, which would not be consistent with PLOS' data policy (<https://journals.plos.org/plosmedicine/s/data-availability>).

**Author response:** We have removed this from the data statement.

**Editor comment 2:** We suggest incorporating "in Australia" in the full title.

**Author response:** We have added, “in New South Wales, Australia” to the title:

- Title, “Opioid agonist treatment and risk of death or rehospitalization following injection drug use-associated bacterial and fungal infections: a cohort study in New South Wales, Australia”

**Editor comment 3:** At line 34 and any similar instances, please make that "39 years". Incidentally, we notice that mean and median ages are both quoted at different points in the ms, and you may wish to make this consistent.

**Author response:** Change made:

- Abstract, line 35: “…mean age 39 years…”

**Editor comment 4:** Around line 41, please adapt the summary of limitations so that this extends to only the final sentence of the "Methods and findings" subsection of the abstract.

**Author response:** Change made so that the summary of limitations is captured within a single, final sentence of this section

- Abstract, Methods and Findings, lines 44-47:

“Study limitations include the use of routinely collected administrative data, which lacks information on other risk factors for injecting-related infections including injecting practices, injection stimulant use, housing status, and access to harm reduction services (e.g. needle exchange, supervised injecting sites); we also lacked information on OAT medication dosages.

**Editor comment 5:** Please avoid "reduced" and "decreased" (risk of death), e.g., at lines 48, 397-399 and 492, in favour of "lower", for example, to avoid implying causality.

**Author response:** Changes made throughout:

- Abstract, Conclusions, lines 49-51:

“Following hospitalizations with injection drug use-associated bacterial and fungal infections, use of OAT is associated with lower risks of death and recurrent injecting-related infections among people with opioid use disorder.”

- Author summary, lines 73-74:

“We found that use of opioid agonist treatment after hospital discharge was associated with both lower risks of death and of rehospitalization with injecting-related infections.”

- Results, lines 338-339:

“Results of survival models are presented in Table 2. In the adjusted model, OAT was associated with lower hazard of all-cause death (adjusted Hazard Ratio [aHR] 0.63, 95% CI 0.57 - 0.70).”

Results, lines 369-370:

- “In the adjusted model, OAT was also associated with lower hazard of rehospitalization (aHR 0.89, 95% CI 0.84 - 0.96; Table 2).”
- Results, lines 396-397:

“In the fully adjusted model in this smaller sample, OAT was also associated with lower hazard of all-cause death (aHR 0.56, 95% CI 0.51 – 0.62).”

- Discussion, lines 403-409:

“Amongst a large cohort of people with opioid use disorder who have been hospitalized with injecting-related bacterial or fungal infections, we found that OAT was associated with lower risk of mortality and of rehospitalization with these infections. Our findings of an association between OAT and lower risk of death among people with opioid use disorder are consistent with prior evidence. The magnitude of the association between OAT and lower rehospitalization risk was more modest, but we are not aware of other interventions shown to reduce risk of reinfection in this setting.”

- Conclusion, lines 497-498:

“Among people with opioid use disorder following hospitalization for injecting-related bacterial or fungal infections, use of OAT is associated with lower risk of death or rehospitalization with injecting-related infections.

**Editor comment 6:** At line 145-146, please substitute the label for the attached checklist.

**Author response:** Change made:

- Methods, lines 149-152:

“This manuscript follows the REporting of studies Conducted using Observational Routinely collected health Data statement for PharmacoEpidemiology (RECORD-PE) guidelines (44) (see S1 RECORD-PE checklist).”

**Editor comment 7:** In the reference list, please abbreviate "Drug Alcohol Depend." consistently.

**Author response:** Changes made.

**Editorial comment 8:** Noting reference 9, please use the journal name abbreviation "PLoS ONE".

**Author response:** Change made.

**Editorial comment 9:** Please remove "[Internet]" from references 46, 57 and any others.

**Author response:** Changes made.

**Editorial comment 10:** Is reference 61 missing the year of publication?

**Author response:** Date of publication added.

We are grateful for your consideration of this paper and feel the reviewers’ suggestions have improved it significantly. We look forward to hearing from you regarding the disposition of the manuscript.

Sincerely, on behalf of all authors,

**Thomas D. Brothers, MD CISAM**

Canadian Institutes of Health Research (CIHR) Fellow

UCL Collaborative Centre for Inclusion Health

University College London

Visiting Fellow

National Drug and Alcohol Research Centre (NDARC)
UNSW – Sydney

Resident Physician (General Internal Medicine & Clinician-Investigator Program)

Department of Medicine
Dalhousie University
